# Editorial Notes

**Published:** 1916-07

**Authors:** 


### Editorial Notes.

**Our Second War Number.**

This issue is our second enlarged and special number, and may be correctly described as a War number, inasmuch as all the original articles have special reference to the War, and, with one exception, by writers who deal with their work in military hospitals at home or abroad. Dr. William Gordon, of Exeter, who is to be congratulated on his re-appointment as President of the Balneological Section of the Royal Society of Medicine, went to Paris and made himself conversant with the valuable work initiated and developed at the Grand Palais Hospital in Paris. We are indebted to him for the results of his personal investigations and observations incorporated in his article in this issue.

Colonel H. L. Norrington, R.A.M.C., has been awarded the D.S.O., a distinction on which the old friends, colleagues and fellow-students of his *Alma Mater* offer him cordial congratulations.

**Captain Orr-Ewing.**

To Captain H. J. Orr-Ewing we offer hearty congratulations on his receiving the Military Cross for good work at the front in France. He is now engaged in one of the French General Hospitals.

